# *Scipion-ED*: a graphical user interface for batch processing and analysis of 3D ED/MicroED data

**DOI:** 10.1107/S1600576722002758

**Published:** 2022-04-22

**Authors:** Viktor E. G. Bengtsson, Laura Pacoste, José Miguel de la Rosa-Trevin, Gerhard Hofer, Xiaodong Zou, Hongyi Xu

**Affiliations:** a Department of Materials and Environmental Chemistry, Stockholm University, Stockholm, SE-106 91, Sweden; b Department of Biochemistry and Biophysics, Science for Life Laboratory, Stockholm University, Stockholm, SE-106 91, Sweden

**Keywords:** electron diffraction, 3D ED, MicroED, data processing, computer programs

## Abstract

Three-dimensional electron diffraction (3D ED)/microcrystal electron diffraction (MicroED) techniques are gaining in popularity. However, the data processing often does not fit existing graphical user interface software, instead requiring the use of the terminal or scripting. *Scipion-ED*, described in this article, provides a graphical user interface and extendable framework for processing of 3D ED/MicroED data. An illustrative project is described, in which multiple 3D ED/MicroED data sets collected on tetragonal lysozyme were processed with *DIALS* through the *Scipion-ED* interface. The ability to resolve unmodelled features in the electrostatic potential map was compared between three strategies for merging data sets.

## Abstract 2

The design and usage of *Scipion-ED*, a graphical user interface program for processing and analysis of three-dimensional electron diffraction (3D ED)/microcrystal electron diffraction (MicroED) data, are presented. A study of the influence of data merging strategies on the ability to resolve unmodelled features of tetragonal lysozyme is included as an illustration of the advantages of *Scipion-ED*.

## 1. Introduction

Over the past few years, three-dimensional electron diffraction (3D ED)/microcrystal electron diffraction (MicroED) techniques for crystal structure determination have gained traction as complementary to X-ray crystallography and cryo-electron microscopy (cryo-EM) (Gemmi *et al.*, 2019 ▸). Structures determined using these techniques include small organic molecules, inorganic materials and proteins (Kolb *et al.*, 2010 ▸; Jiang *et al.*, 2011 ▸; Gorelik *et al.*, 2012 ▸; Martínez-Franco *et al.*, 2013 ▸; Smeets *et al.*, 2013 ▸, 2014 ▸; Zhang *et al.*, 2013 ▸; Shi *et al.*, 2013 ▸; Su *et al.*, 2014 ▸; Yonekura *et al.*, 2015 ▸; Clabbers *et al.*, 2017 ▸, 2021 ▸; Gruene *et al.*, 2018 ▸; Jones *et al.*, 2018 ▸; Lanza *et al.*, 2019 ▸; Xu *et al.*, 2019 ▸). Among the more recent improvements in 3D ED/MicroED are the use of continuous rotation of the crystal during data collection, commonly referred to as MicroED (Nannenga & Gonen, 2016 ▸) or continuous rotation electron diffraction (cRED) (Wang *et al.*, 2018 ▸), and the use of fast and sensitive hybrid pixel detectors (van Genderen *et al.*, 2016 ▸; Llopart *et al.*, 2007 ▸). These improvements have enabled rapid acquisition of 3D ED/MicroED data and the possibility of processing those data using software developed for X-ray crystallography, such as *XDS* (Kabsch, 2010 ▸) and *DIALS* (Winter *et al.*, 2018 ▸).

The increasing acquisition speed and the increasing adoption of 3D ED/MicroED naturally lead to larger quantities of data that need to be processed and analysed as well as an increasing number of scientists at the novice level for the specific data processing methods. An increased quantity of data is also the result of data collection automation (Wang *et al.*, 2019 ▸). Consequently, tools are needed to process the data more easily and more quickly in a straightforward way. An initial solution has been suggested through the program *edtools*, which adds a layer for running *XDS* on multiple data sets at once and then merging those with sufficient similarity (Wang *et al.*, 2019 ▸).

The challenge of large quantities of data has been handled in X-ray crystallography through integrated pipelines with graphical user interfaces (GUIs). Examples include *CCP4i2* (Potterton *et al.*, 2018 ▸), *XDSGUI* (Kabsch, 2016 ▸) and *KAMO* (Yamashita *et al.*, 2018 ▸). However, these interfaces are typically designed for synchrotron beamlines where the experimental geometry is well defined, image formats are standardized and the recorded diffraction patterns are from a highly curved Ewald sphere. These criteria are often not met in the case of electron diffraction. Data processing parameters need to be tailored for different microscope and detector setups. In addition, the collection of 3D ED/MicroED data using cRED sometimes involves defocusing the beam to create an image instead of the diffraction pattern, which requires their exclusion during processing (Smeets *et al.*, 2017 ▸; Cichocka *et al.*, 2018 ▸; Wang *et al.*, 2019 ▸). Existing GUIs such as those listed do not provide access to all the necessary parameters from electron diffraction data.

Large quantities of data and a need for integrated workflows are also found in cryo-EM. One solution designed for those needs is *Scipion* (de la Rosa-Trevín *et al.*, 2016 ▸). It provides a workflow-focused GUI where each step is represented by a protocol. The protocols offer a unified interface for different programs used for processing cryo-EM micrographs. *Scipion* also stores metadata such as the location of raw images and the input parameters needed for repeating a certain protocol or a workflow consisting of multiple linked protocols. It is thus possible to reuse the same parameters for a different project and to deposit the processing parameters along with the raw data in online databases. It is also possible to add new protocols when additional programs become available, using the same interface and storage formats for input parameters and output. Furthermore, the framework is suitable for developing additional interfaces on top of the underlying infrastructure. For example, some cryo-EM processing workflows were exposed as web tools (Mingo *et al.*, 2018 ▸) for easy access and learning. A lot of effort has also been dedicated to on-the-fly data processing (Gómez-Blanco *et al.*, 2018 ▸), allowing overlapping data collection with data pre-processing and quick quality assessment.

This article describes *Scipion-ED*, which extends the *Scipion* framework to include 3D ED/MicroED processing. This GUI will be useful for existing and new 3D ED/MicroED users, especially the facilities and users that employ both 3D ED/MicroED and cryo-EM on the same equipment, since requirements for training and software maintenance are reduced. There are a number of advantages for using *Scipion-ED* to process 3D ED/MicroED data. Firstly, graphical user interfaces are often preferable to the command line when first using a program, making it more user friendly for beginners. Secondly, copying and pasting a workflow is more straightforward than writing a script when reusing processing parameters for a new data set. It allows large quantities of data to be processed rapidly. Furthermore, *Scipion-ED* offers convenience in data analysis and developing data merging strategies. Finally, it is designed in a way that prevents accidental overwriting of the output from one run by the next. Such accidents are most likely when performing initial exploratory analysis or when reprocessing an old data set.

In this paper, we describe the implementation of *Scipion-ED* and demonstrate how it can be used for batch data processing, data analyses, improving data processing and finding merging strategies.

## 2. Scipion-ED

**Figure 1:**
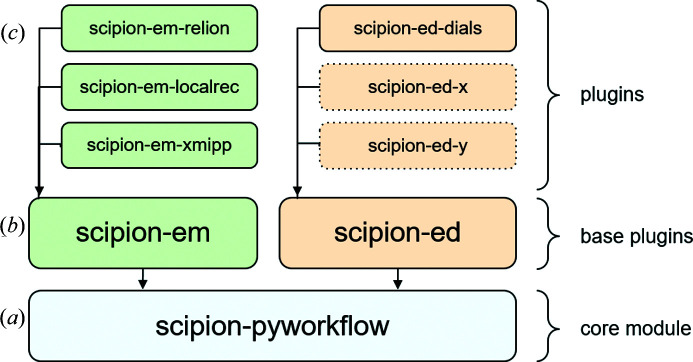
Basic structure of the *Scipion* software. The package *scipion-pyworkflow* contains all the elements common to all versions of *Scipion*. Base plugins are then added for electron microscopy and electron diffraction, defining which information to store for micrographs and diffraction patterns, respectively, as well as defining some basic protocols. Additional protocols are then added as part of the plugins that interface with external software. In the case of *scipion-ed-dials*, the protocols create command line instructions for *DIALS*, which are then run automatically. Additional software can be added to the platform in the future, as indicated by *scipion-ed-x* and *scipion-ed-y*.

*Scipion* is a framework built from multiple Python modules that define the basic workflow engine and provide the protocols that can be executed. The common components for defining the GUI, keeping track of metadata in a database and handling of job execution are implemented in the *scipion-pyworkflow* module [Fig. 1 ▸(*a*)]. Specific protocols and input/output data entities for a given scientific domain are defined on top of the core module. Currently, the most developed domain is cryo-EM for which *scipion-em* serves as the base plugin [Fig. 1 ▸(*b*)]. Several plugins have been implemented for integrating cryo-EM software packages into *Scipion*, such as *relion* (Sharov *et al.*, 2021 ▸), *localrec* (Abrishami *et al.*, 2021 ▸) and *xmipp* (Sorzano *et al.*, 2004 ▸) [see Fig. 1 ▸(*c*)]. The base plugin of *Scipion-ED* thus provides the interface between diffraction-oriented protocols and *scipion-pyworkflow* as well as basic protocols for processing workflows, such as importing diffraction patterns. Specific protocols for interfacing with the actual data processing software are built on top of the base plugin.

## 3. Graphical user interface and *scipion-ed-dials*

Each protocol in *scipion-ed-dials* represents a processing step in *DIALS*. The choice of *DIALS* was made because both *Scipion* and *DIALS *are Python based and open source. This offers future potential of calling internal *DIALS* functions directly from *scipion-ed-dials*, rather than through a shell.

**Figure 2:**
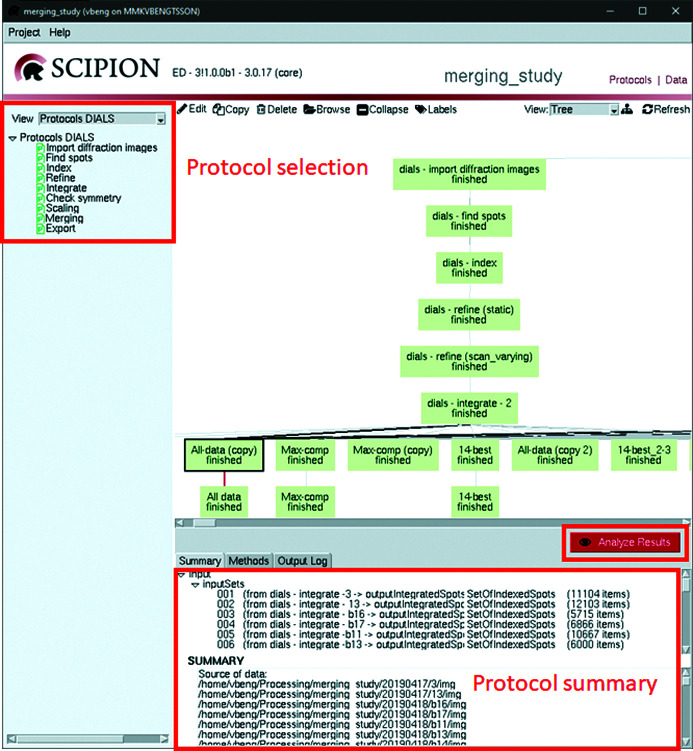
The main interface of *Scipion-ED*. Protocols can be selected from the menu on the left. All runs are displayed as the boxes in the central window, and a summary of the selected protocol’s output is shown at the bottom. The ‘Methods’ and ‘Summary’ tabs in the same area can be used to see citation information and the full terminal output, respectively. Inputs to a selected protocol can be edited after double-clicking to open the input window as shown in Fig. 3 ▸, while the button ‘Analyze Results’ opens a window like that shown in Fig. 4.

The full data processing, from importing of diffraction patterns to exporting of a scaled reflection file, is illustrated in Fig. 2 ▸. Each step of the process is described below, with a general description and specific examples of the processing of data sets collected on tetragonal lysozyme crystals. The supplementary information contains details of the data collection.

Information moves between protocols as shown by the lines connecting the green boxes, using a database and Python objects named Sets. The Sets are Python classes defined in the base plugin of *Scipion-ED* and contain metadata about multiple items of the same type. As an illustration, the import protocol generates a DiffractionImage object for each imported diffraction pattern, containing information about file location and information extracted from the file header. SetOfDiffractionImages represents all the DiffractionImages related to a run of the import protocol and also includes information on which images to skip (if any; see *Importing data* [Sec sec4] below).

Additional protocols can be added to the process from the menu on the left side of the interface or by copying the selected protocol. The bottom of the screen shows a summary of the input given to and actions performed by the protocol. The red button labelled ‘Analyze Results’ gives access to additional information and analysis tools implemented in *DIALS*.

## 4. Importing data

**Figure 3:**
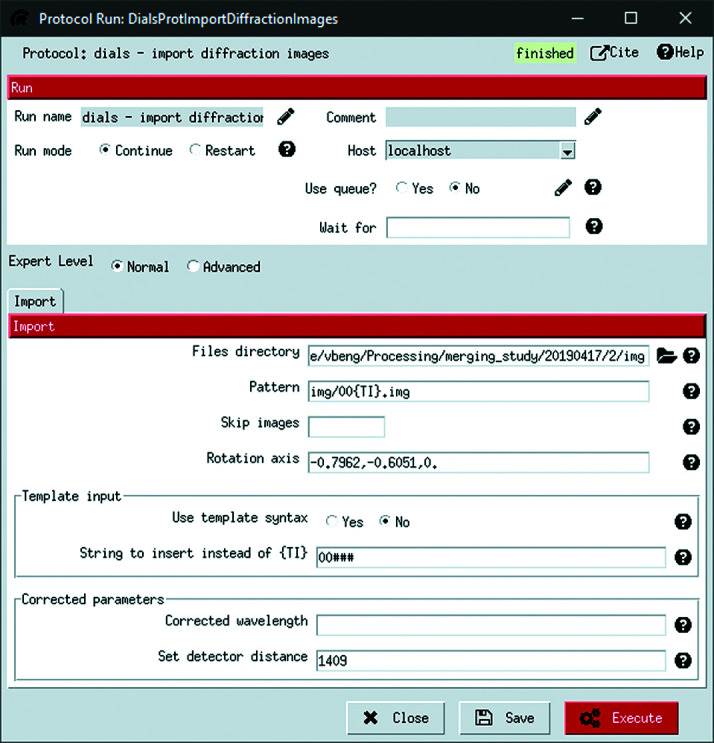
The interface for running *dials.import*. The ‘Files directory’ is the path to the data for the unique crystal, while ‘Pattern’ indicates the subdirectories and file patterns that are common to all data sets. Boxes are included for overwriting parameters such as rotation axis and detector distance. Access to overwriting the rotation axis is provided by first selecting ‘Expert Level: Advanced’.

Specifying the location of the diffraction images is a necessary step common to all data reduction programs. The input interface shown in Fig. 3 ▸ is therefore designed in the base *Scipion-ED* plugin. Locating the diffraction images is split into the ‘Files directory’ input, where a browser GUI can be used to identify the unique part of a directory path, and the ‘Pattern’ box, where the general structure of subdirectories and filename can be specified.

The ‘{TI}’ placeholder replaces digits in the filenames that uniquely identify a specific diffraction pattern within the folder. The ‘Skip images’ option can then be used to mark every *n*th frame (with *n* used as input) as inappropriate for use, such as when using defocused images in *Instamatic* (Cichocka *et al.*, 2018 ▸; Smeets *et al.*, 2021 ▸). The empty boxes in Fig. 3 ▸, as well as in other protocols, indicate that by default no value is used. Other boxes have pre-filled values. Some of these are based on *DIALS* defaults, such as the number of processes to use (see Fig. 6 in Section 6). Others, such as the insertion string for import templates (Fig. 3 ▸), are based on our experience. These defaults are intended as a starting point, and fine-tuning for a particular experiment might be required.

The plugins define how the input is used. For *scipion-ed-dials*, the plugin creates a text string with the full command line call to run *dials.import*, the corresponding step in *DIALS*. The typical behaviour in *Scipion-ED* is that each diffraction pattern is added to the call, but it is also possible to use a template function instead. The metadata contained in the first image of the series will then be extrapolated to the entire series. A use case for the template is where the goniometer rotation (PHI in Fig. S1) is set to the same value for all diffraction images instead of the true angle.

The lysozyme data set (Xu & Bengtsson, 2022 ▸) used in this work as an example was imported as SMV files (described in the supplementary information), but other formats can also be used in *DIALS* [as described by Parkhurst *et al.* (2014 ▸)]. However, the converted patterns are rotated relative to the detector format expected in *DIALS*. The goniometer rotation axis therefore had to be corrected using the dedicated boxes. Initial access to the rotation axis box requires the ‘Expert Level’ to be set as ‘Advanced’. Details on how to identify the correct rotation axis can be found in the *DIALS* tutorial *MyD88^TIR^ small wedges* (Waterman, 2021 ▸). It is also possible to use the program *dials.find_rotation_axis*, added to *DIALS* in version 3.7.0. The rotation axis should be constant for each experimental setup.

## 5. Spot finding

**Figure 4:**
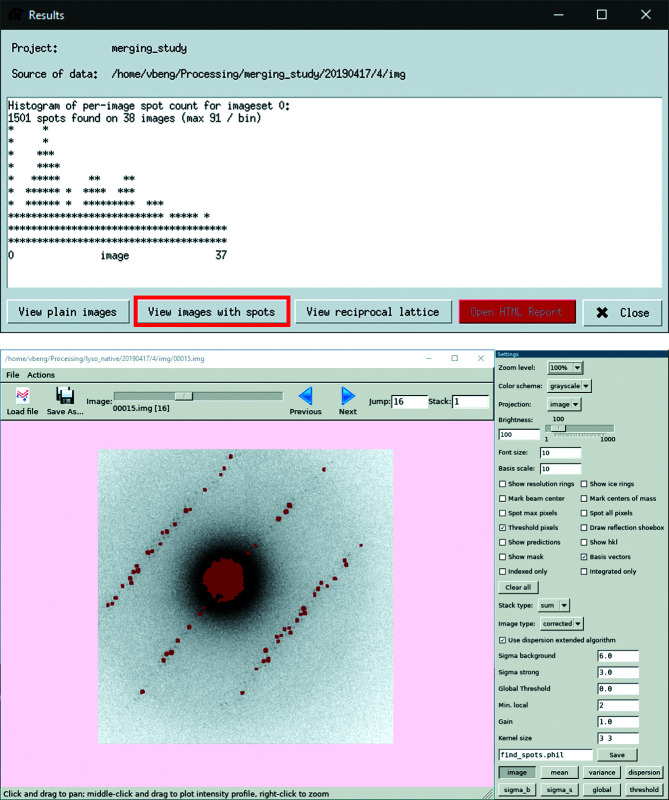
Result window of the spot-finding protocol (top) with highlight on the button to access the *DIALS* image viewer (bottom). Through the *DIALS* image viewer the impact of the spot-finding parameters can be further examined to optimize the spot finding and resolve issues related to indexing. The content of the results view is extracted from log files, and the red button will open any available HTML report.

Spot finding is the step in *DIALS* where groups of pixels that have detected a signal are identified and assigned as a spot that can be indexed in later steps. The resolution range 2.0–30.0 Å was used in the processing of the lysozyme data sets to avoid noise close to the direct beam and at higher resolutions. It is also possible to select a range of images to include for processing. The most common reason to do this is to remove frames where the crystal is known to be outside the beam. There are also options available for adjusting the settings of the spot-finding algorithm. These include a choice of which specific algorithm to use and how much a strong spot should deviate from the background. The effects of changing a parameter can be tested through the *DIALS* image viewer, as illustrated in Fig. 4 ▸. The figure contains both the spot-finding results viewer, with buttons for opening additional viewers, and the image viewer.

Out of 47 lysozyme data sets, 33 could be fully processed with no changes to the default parameters beyond the resolution ranges. Two additional data sets could be used after further optimization of the spot-finding settings.

**Figure 5:**
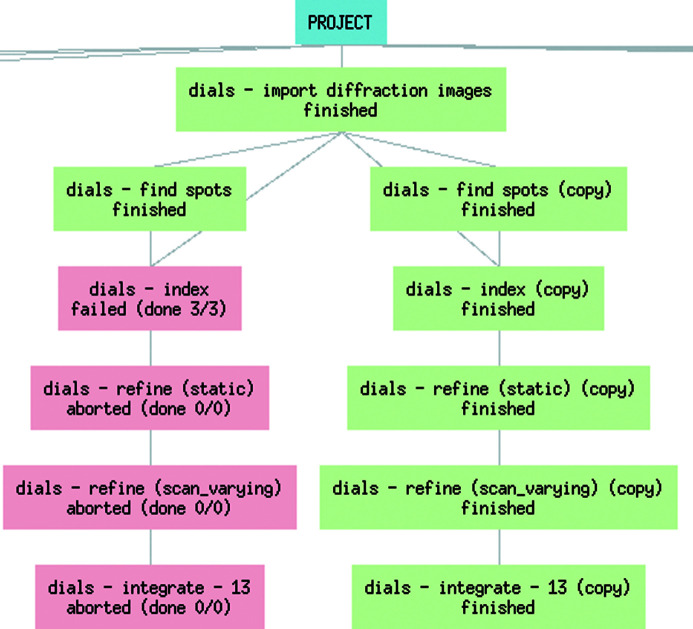
Parallel workflow created with alternative input parameters (sigma strong) for the spot finding until suitable parameters for processing the data are found.

The results of multiple different combinations of input parameter values had to be compared for the data sets that could not be processed using default ED settings for all protocols. *Scipion-ED* greatly simplified this process by allowing simple copying of workflows, making it easy to change parameter values and ensuring that the original processing flow is kept for comparison. Identifying where changes were introduced and which step fails is also easy, as exemplified by the split tree and failed protocols in Fig. 5 ▸. Using scripts or pure command line input to accomplish the same comparison would require more steps and risk overwriting the original processing, and it would be less obvious as to where changes were introduced.

## 6. Indexing and refinement

**Figure 6:**
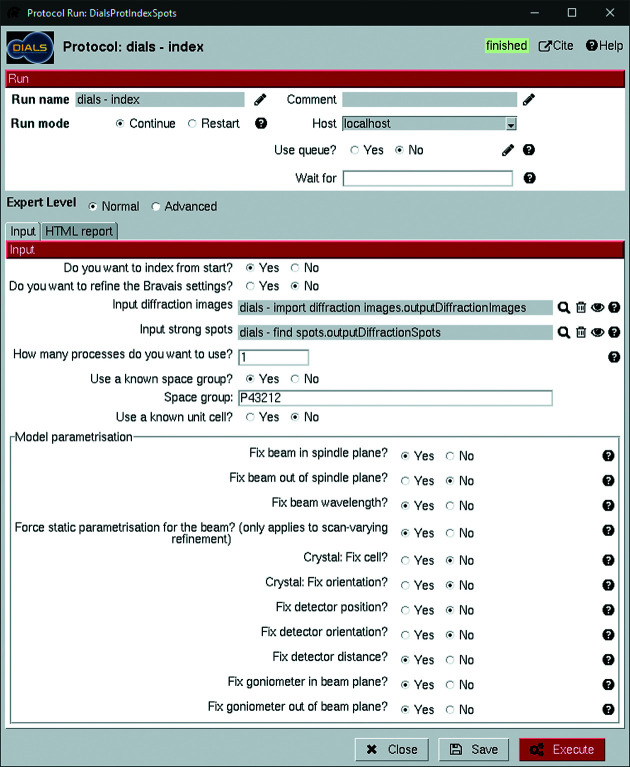
Indexing protocol input interface. The model parameterization influences which parts of the experimental model to refine and is the same as in the refinement protocol (Fig. 7). It is also possible to add refinement of the Bravais setting, use a known space group or index with a known unit cell. All settings except the usage of a known space group are set to the default values, where only the fixing of the detector distance deviates from the default value in *DIALS*.

**Figure 7:**
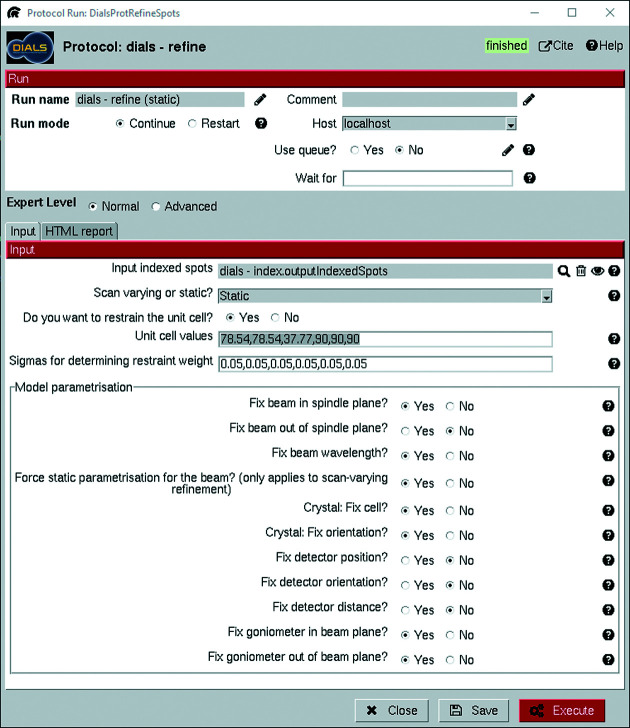
Input for static refinement. The original work was done using v3.0.1 of *scipion-ed-dials*, while the interface depicted is from v3!1.0.0b2. The refinement is tied to unit-cell parameters. These were given in restraints.phil, located in the *Scipion-ED* project directory in the original work. The updated interface depicted instead provides boxes to enter the relevant values, and *Scipion-ED* creates the phil file as needed. Another change between versions is that a binary choice of static or scan varying has been expanded to include ‘Auto’ and ‘DIALS default’ in the drop-down menu.

The indexing protocol incorporates three functions: indexing, refining the Bravais settings and reindexing based on the refined settings. The first two of these also include some degree of refinement of the experiment model, although refinement is primarily performed by *dials.refine* through the refinement protocol. The input interfaces for both the indexing protocol (Fig. 6 ▸) and the refinement protocol (Fig. 7 ▸) contain options to fix parameters of the diffraction geometry and the crystal models as part of the refinement. It is important to fix either the detector distance or the unit-cell dimensions in electron diffraction, since these will influence each other owing to the flatness of the Ewald sphere. The default option in *scipion-ed-dials* is to keep the detector distance fixed.

The current implementation of *scipion-ed-dials* always returns the highest symmetry suggested from refining the Bravais setting, since this is often a reasonable assumption. Trying other symmetries can be done by rerunning the indexing step with a given space group, as shown in Fig. 6 ▸. The corresponding model file will be automatically used for output from the protocol, but reindexing the reflection file requires additional confirmation through checkboxes.

One of the primary considerations during cell refinement is whether to use static or scan-varying refinement. The difference is that scan-varying refinement allows some parameters to vary throughout the data set, such as orientation and unit cell (Winter *et al.*, 2018 ▸). For the lysozyme data set, static refinement was performed first with the unit-cell parameters restrained to values found in the literature [comparable to the process used by Waterman (2021 ▸)] to provide a good starting point, followed by scan-varying refinement to improve the model. One round of static refinement followed by scan-varying refinement has become standard in *dials.refine* after the design of the refinement protocol used during processing. The corresponding changes have been added to version 3!1.0.1 of *scipion-ed-dials*.

The restraints given during the static refinement were provided through a phil-format file (included in the supplementary information) in the project directory and given as direct command line input. A field for such input is provided in each protocol by selecting the advanced expert level. Anything written there, such as additional flags, will be added to the end of the command line call to *DIALS*. Therefore, parameters that have not been added to the *scipion-ed-dials* interface can still be used in the *DIALS* processing.

Avoiding rarely used parameters cluttering the interface is the primary reason for parameter exclusion. Moreover, recently added parameters in *DIALS* may not yet be included in *scipion-ed-dials*. Thus, the requirement to write a phil file with the restraints has been removed in version 3!1.0.1 of *scipion-ed-dials*. The values can now instead be added directly in dedicated boxes, as seen in Fig. 7 ▸.

## 7. Integration, scaling and export

Integration of all intensity belonging to the same indexed spot from multiple diffraction images in the refined data sets is typically straightforward, with or without trimming the resolution range being the main question. Trimming the resolution reduces computational time, but excessive trimming removes useful information. Trimming is also possible during scaling (or earlier during spot finding), but the default is to keep everything. A resolution range of 2.0–30.0 Å was kept for all protocols during the processing of the lysozyme data sets.

**Figure 8:**
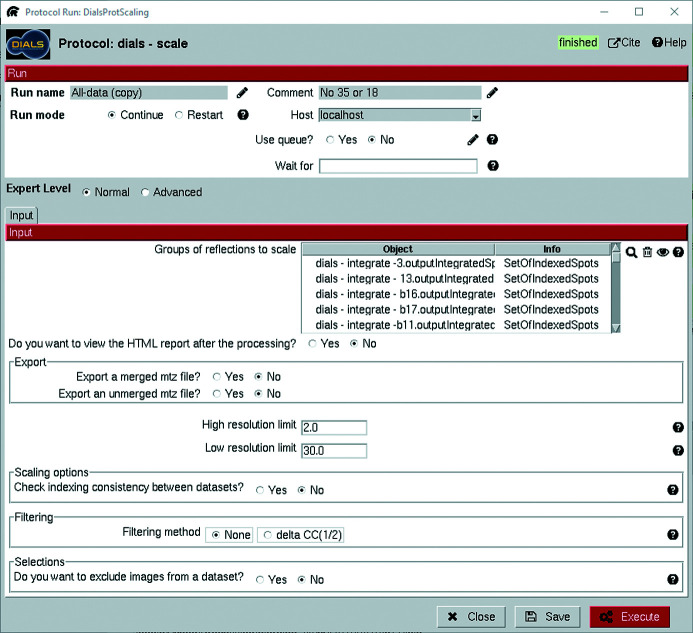
Input interface for scaling. The option to use multiple input sets is the main difference compared with previous protocols.

Scaling offers more options, as seen in Fig. 8 ▸. The most obvious difference compared with other protocols is that scaling accepts the output from multiple data sets as input. They can be selected from a list in the GUI, while the equivalent operation using the command line would require the entering of each file path individually or through regular expressions. That is one reason for why *Scipion-ED* is well suited to the comparison of strategies for merging data sets – the same scaling protocol could be copied multiple times and different selections of data sets could be compared.

Another significant difference between scaling and other protocols is that scaling creates an HTML summary automatically, rather than requiring *dials.report* to run. In protocols other than scaling, there is therefore a tab marked ‘HTML report’, and all interfaces allow the option to open the file as soon as the protocol has finished. It is also always possible to open the report through the results viewer (red button in Fig. 4 ▸), and the report will be opened in a web browser or other default program for opening HTML files. In particular, the scaling report provides a useful visual presentation of the results, such as which frames contribute to poor signal-to-noise ratios or which resolution is appropriate. Such frames can then be excluded by giving their indices in the interface.

Excluding images, as enabled by the ‘Selections’ section at the bottom of Fig. 8 ▸, is also very useful for removing frames that are known to contain unreliable intensities. Typical examples are frames collected along major zone axes of the crystal, causing strong dynamical effects, and frames collected when the crystal moved out of the electron beam.

There are useful ways of removing poor diffraction patterns, groups of diffraction patterns or even data sets before analysing the report. Checking the indexing consistency runs *dials.cosym* as part of the scaling, using an algorithm for Patterson group symmetry determination for cases with indexing ambiguity (Gildea & Winter, 2018 ▸). Similarly, the filtering that can be accessed through the GUI allows the automatic removal of data sets or frames that would significantly reduce the CC_1/2_ of the overall data set. A protocol for running *dials.symmetry* to identify the Laue group symmetry before scaling has also been added to *scipion-ed-dials* in version 3!1.0.1. Future work includes adding full support for these steps through the *xia2.multiplex* tool.

Important indicators for judging the data quality can be found in the result viewer as well as in the HTML file, with the latter providing more attractive formatting. The primary quality indicators are CC_1/2_, *I*/σ(*I*), *R*_meas_, *R*_pim_ (Karplus & Diederichs, 2015 ▸) and completeness. Statistics for each resolution shell as well as the overall statistics are presented both in the results viewer and in the HTML output.

Finally, exporting to other file formats for further processing outside the *Scipion-ED* pipeline is possible, with available parameters determined by the desired file format.

## 8. Illustrative example of *Scipion-ED* data processing

The processing of the tetragonal lysozyme data sets used as examples in the description above was carried out to compare merging strategies regarding the structure solution and refinement of tetragonal lysozyme (space group *P*4_3_2_1_2, No. 96). Two of the 47 data sets were used for initial determination of suitable parameters, such as the rotation axis and detector distance used in the import step and resolution ranges used in spot finding. The resulting workflow, from importing to scaling of individual data sets, was then copied and reused for the remaining 45 data sets.

Thirty-five data sets could be processed in this way, either directly (33 data sets) or after modifications of the spot-finding parameters as described above. Five of the remaining 12 data sets could not be imported at all, while seven were found to have inherent problems even after optimization and were eventually discarded. The failure to import images is presumed to be the result of file corruption during conversion, while those that could not be used even after optimization were probably subject to beam damage.

Two strategies were devised for the merging of data sets. In strategy 1, data sets with the most favourable overall merging statistics with regard to the three criteria CC_1/2_ > 0.8, *I*/σ(*I*) > 2 and *R*_meas_ < 0.60 were merged. Overall values were selected because they indicate the average data quality, with the specific numbers chosen according to experience from similar data sets. The data sets were selected manually on the basis of inspection of the output. In strategy 2, all data sets were merged using a 2.0 Å resolution cut-off, emulating a strategy where no particular consideration is given to the choice of data sets for merging. A scaled and merged mtz file for each strategy was exported for further structure solution and refinement. Final overall statistics are presented in Table 1 ▸. The lack of completeness despite 25-fold and 43-fold multiplicity could be the result of preferred orientation.

**Figure 9:**
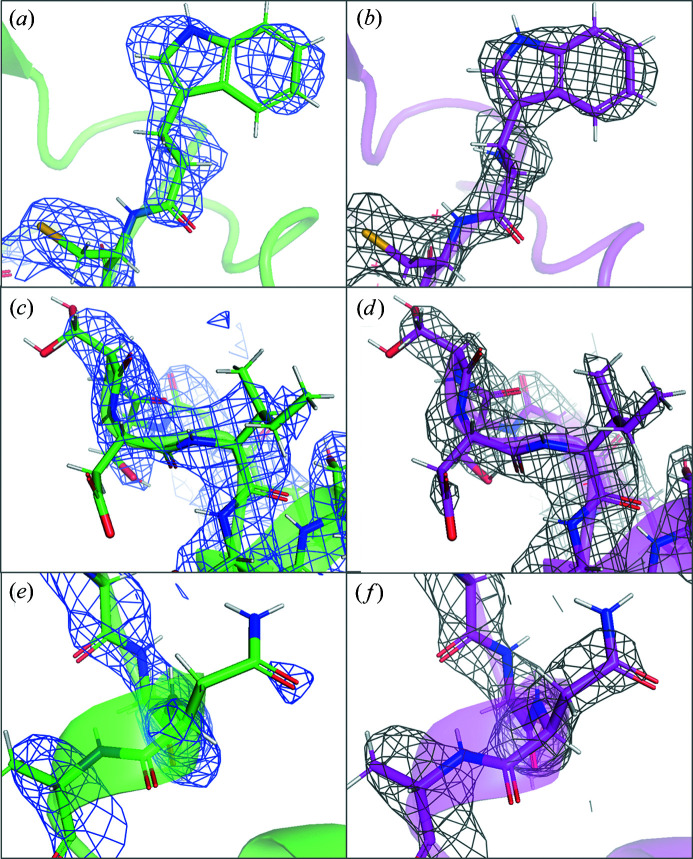
Comparison of the models and 2*F*_o_–*F*_c_ electrostatic potential map obtained for strategy 1 (green model, blue isomesh) and strategy 2 (magenta model, grey isomesh) for different parts of the model: (*a*), (*b*) the Trp63 residue, (*c*), (*d*) Ise88 and Asp87, and (*e*), (*f*) Asn106. All maps are contoured to 1.5σ.

Molecular replacement based on the lysozyme structure PDB 193l (Vaney *et al.*, 1996 ▸) was performed through *Phaser* (McCoy *et al.*, 2007 ▸) to obtain an initial model for each strategy. Refinement was performed in *phenix.refine* (Afonine *et al.*, 2012 ▸) with five refinement cycles, grouped *B* factors and a resolution cut-off at 2.6 Å. The final model was validated using *MolProbity* through the *Phenix* suite (Chen *et al.*, 2010 ▸; Williams *et al.*, 2018 ▸). Strategy 1 had the lowest completeness (73.4%) but the highest overall CC_1/2_ (0.921) and a 2.610 Å resolution based on CC_1/2_ > 0.330, while strategy 2 had 2.987 Å resolution. At the same time, strategy 2 resulted in a higher overall completeness of 86.9%. We found that, despite the differences in completeness, strategy 1 resulted in a slightly lower *R*_work_/*R*_free_. However, a higher number of ordered solvent molecules could be modelled in the map obtained from strategy 2 compared with strategy 1 (13 compared with two water molecules respectively; Table 2 ▸). Moreover, the electrostatic potential map for strategy 2 has residue side chains that are better defined and improved continuity along the peptide backbone compared with strategy 1 (Fig. 9 ▸). These results indicate that merging all data sets is advantageous for resolving more details in the electrostatic potential map, despite the reduced data quality of the high-resolution reflections. This is in agreement with previously reported studies, where increased redundancy resulted in a more detailed model (Xu *et al.*, 2018 ▸).

## 9. Conclusions

*Scipion* is a versatile tool for data processing, with clear visualization and simple sharing of workflows. These strengths have been brought to 3D ED through the *Scipion-ED* base and *scipion-ed-dials* plugins. Novice users can benefit from the presentation of common parameters, while experienced users still have access to all parameters found through the command line. All users can benefit from simple reuse of successful workflows and comparing the results of different processing. Such a comparison was made between two different strategies for merging data, where more details could be resolved in the electrostatic potential map by using all available data instead of only the highest quality data.

## 10. Availability

The *scipion-ed-dials* module depends on having *DIALS* available in the path. The most recent version can be found from the *DIALS* web site at https://dials.github.io/installation.html. Installing the *Scipion-ED* base plugin and *scipion-ed-dials* also requires a Python 3 environment with pip, such as one set up with Anaconda or Miniconda. Python 3.8 is recommended. In that environment, installation is done with the one line pip install scipion-ed scipion-ed-dials. All dependencies except *DIALS* itself will then automatically be installed as well. The program is open source under a GPL-3.0 licence, and the source code for *Scipion-ED* and plugins can be found at https://github.com/scipion-ed. Contributions from the community such as bug reports, fixes, suggestions and improvements are welcome.

The raw data used for the examples are available at https://doi.org/10.5281/zenodo.6037632.

## Supplementary Material

Supporting information file. DOI: 10.1107/S1600576722002758/yr5085sup1.pdf

Raw data used in processing: https://doi.org/10.5281/zenodo.6037632

## Figures and Tables

**Table 1:** Reflection statistics for the two different merging strategies

Parameters	Strategy 1: best statistics	Strategy 2: all data sets
Space group	*P*4_3_2_1_2	*P*4_3_2_1_2
Cell dimensions		
*a*, *b*, *c* (Å)	78.93, 78.93, 36.96	78.75, 78.75, 37.33
α, β, γ (°)	90, 90, 90	90, 90, 90
Resolution range (Å)	27.9–2.0	27.8–2.0
High-resolution cut-off† (Å)	2.6	3.0
*R*_meas_	0.627	1.192
*R*_pim_	0.110	0.165
Mean *I*/σ(*I*)	6.5	7.7
CC_1/2_	0.921	0.847
Completeness (%)	73.40	86.90
Multiplicity	25.4	43.4

† Recommended high-resolution cut-off based on CC_1/2_ > 0.33 as suggested by *DIALS*, rounded to one decimal place.

**Table 2:** Validation statistics for the final structure refinement

Refinement validation	Strategy 1	Strategy 2
Resolution (Å)	27.9–2.6	27.8–2.6
No. of reflections (total/unique)	2605/100 066	3323/200 258
*R*_work_/*R*_free_	0.243/0.273	0.252/0.281

No. of atoms		
Protein	1965	1965
Ligand/ion	0	0
Water	2	13

*B* factor (Å^2^)		
Protein	23.86	21.36
Ligand/ion	N/A	N/A
Water	8.76	21.12

R.m.s. deviations		
Bond lengths (Å)	0.0045	0.0038
Bond angles (°)	0.66	0.68

Ramachandran		
Favoured (%)	92.13	92.91
Allowed (%)	7.87	7.09
Outliers (%)	0.00	0.00
Clashscore	7.63	6.11
Rotamer outliers (%)	0.94	0.00
