# Supplementary material for: Scipion-ED: a graphical user interface for batch processing and analysis of 3D ED/MicroED data
Source: J Appl Crystallogr. 2022 Apr 22;55(Pt 3):638–46. doi: 10.1107/S1600576722002758 (PMC9172039; doi:10.1107/S1600576722002758)
Supplement: Supplementary file 1 [file j-55-00638-sup1.pdf]

JOURNAL OF
APPLIED
CRYSTALLOGRAPHY

Volume 55 (2022)

Supporting information for article:

Scipion-ED — a graphical user interface for batch processing and analysis of 3D ED/MicroED data

Viktor E.G. Bengtsson, Laura Pacoste, José Miguel de la Rosa-Trevin, Gerhard Hofer, Xiaodong Zou and Hongyi Xu

S1. Data collection and file format conversion

The data were collected on lysozyme microcrystals using a JEOL 2100 transmission electron microscope (TEM) with a LaB6 filament operating at 200 kV and equipped with a TimePix hybrid-pixel detector. The detector was controlled through the SoPhy software provided by Amsterdam Scientific Instruments along with the camera.

A script was used to convert the images to SMV format, integrating certain metadata into the file header as shown in Figure S 1 (left). Of particular note are the BEAMLINE and DISTANCE parameters in the file header. Here, TIMEPIX_SU indicates the specific setup used and allows DIALS to load default values for the goniometer rotation axis and other instrument settings. The distance is based on the nominal camera length of the microscope and converted using a table included in the script.

If no specific setup is indicated (BEAMLINE parameter) such as in Figure S1 (right), then other default values will be used or specific information can be included during the import step.

S2. PHIL file for the restraints

The restraints.phil file used in processing had the following contents:

```
refinement
{
  parameterisation
  {
    crystal
    {
      unit_cell
      {
        restraints
        {
          tie_to_target
          {
            values=78.54,78.54,37.77,90,90,90
            sigmas=0.05,0.05,0.05,0.05,0.05,0.05
          }
        }
      }
    }
  }
}
```

```
{
HEADER_BYTES=  512;
DIM=2;
BYTE_ORDER=little_endian;
TYPE=unsigned_short;
SIZE1=516;
SIZE2=516;
PIXEL_SIZE=0.055000;
BIN=1x1;
BIN_TYPE=HW;
ADC=fast;
CREV=1;
BEAMLINE=TIMEPIX_SU;
DETECTOR_SN=901;
DATE=Tue Jun 26 09:43:09 2007;
TIME=0.096288;
DISTANCE=1480.56;
TWOTHETA=0.00;
PHI=20.00;
OSC_START=20.00;
OSC_RANGE=0.90;
WAVELENGTH=0.025100;
BEAM_CENTER_X=226.75;
BEAM_CENTER_Y=233.23;
DENZO_X_BEAM=12.83;
DENZO_Y_BEAM=12.47;
}
```

```
{
HEADER_BYTES=512;
DIM=2;
BYTE_ORDER=little_endian;
TYPE=unsigned_short;
SIZE1=4096;
SIZE2=4096;
PIXEL_SIZE=0.014;
BIN=1x1;
DETECTOR_SN=unknown;
DATE=Mon Feb 1 19:10:02 2021;
TIME=0.5;
DISTANCE=4294.150054;
TWOTHETA=0;
PHI=-34.513568;
OSC_START=-34.513568;
OSC_RANGE=0.5;
WAVELENGTH=0.019691;
BEAM_CENTER_X=28.672;
BEAM_CENTER_Y=28.672;
}
```

Figure S1 Example of two SMV headers from a JEOL 2100 LaB6 with a TimePix detector (left) and a Titan Krios with a CETA-D detector (right) as viewed in Notepad ++. The SIZE1 and SIZE2 keywords indicate the number of pixels in the x and y directions, and PIXEL_SIZE is the physical size of each pixel in millimetres. TIME is the exposure time of each diffraction image in seconds and DISTANCE the camera length in millimetres. PHI and OSC_START will have the same value for a single axis goniometer in a microscope, and indicates the rotation angle of the goniometer at the start of the frame's exposure. OSC_RANGE is the angle in degrees that the goniometer rotates during the collection of the frame. WAVELENGTH depends on the acceleration voltage and is given in Ångström. BEAM_CENTER_X and BEAM_CENTER_Y indicate the location of the central beam on the detector. In the left image, they are given in pixels, while the right image gives the position in millimetres. Both values should be in millimetres. A correction is made through a built-in format class in DIALS, designed specifically for the instrument indicated by the line BEAMLINE=TIMEPIX_SU. The same format class also provides a default value for the goniometer axis and other instrument constants. The rest of the parameters are either not needed or indicate how to decode the binary data.
